# Effects of resistance training and turmeric supplementation on reactive species marker stress in diabetic rats

**DOI:** 10.1186/s13102-020-00194-9

**Published:** 2020-08-06

**Authors:** Ailton Santos Sena Júnior, Felipe José Aidar, Jymmys Lopes Dos Santos, Charles Dos Santos Estevam, Jessica Denielle Matos dos Santos, Ana Mara de Oliveira e Silva, Fábio Bessa Lima, Silvan Silva De Araújo, Anderson Carlos Marçal

**Affiliations:** 1 Department of Physical Education, Universidade Federal de Sergipe, São Cristóvão, Sergipe Brazil; 2 Group of Studies and Research of Performance, Sport, Health and Paralympic Sports – GEPEPS, Universidade Federal de Sergipe, São Cristóvão, Sergipe Brazil; 3 Department of Physiology, Universidade Federal de Sergipe, São Cristóvão, Sergipe Brazil; 4 Department of Nutrition, Universidade Federal de Sergipe, São Cristóvão, Sergipe Brazil; 5 Department of Physiology and Biophysics, Universidade de São Paulo, São Paulo, Brazil; 6 Department of Morphology, Universidade Federal de Sergipe, São Cristóvão, Sergipe Brazil

**Keywords:** Turmeric, Resistance training, Diabetes mellitus

## Abstract

**Background:**

Type 1 *diabetes mellitus* (T1DM) is a metabolic disease characterized by hyperglycemia and excessive generation of reactive oxygen species caused by autoimmune destruction of beta-cells in the pancreas. Among the antioxidant compounds, *Curcuma longa* (CL) has potential antioxidant effects and may improve hyperglycemia in uncontrolled T1DM/TD1, as well as prevent its complications (higher costs for the maintenance of health per patient, functional disability, cardiovascular disease, and metabolic damage). In addition to the use of compounds to attenuate the effects triggered by diabetes, physical exercise is also essential for glycemic control and the maintenance of skeletal muscles. Our objective is to evaluate the effects of CL supplementation associated with moderate- to high-intensity resistance training on the parameters of body weight recovery, glycemic control, reactive species markers, and tissue damage in rats with T1DM/TD1.

**Methods:**

Forty male 3-month-old Wistar rats (200–250 g) with alloxan-induced T1DM were divided into 4 groups (*n* = 7–10): sedentary diabetics (DC); diabetic rats that underwent a 4-week resistance training protocol (TD); CL-supplemented diabetic rats (200 mg/kg body weight, 3x a week) (SD); and supplemented diabetic rats under the same conditions as above and submitted to training (TSD). Body weight, blood glucose, and the following biochemical markers were analyzed: lipid profile, aspartate aminotransferase (AST), alanine aminotransferase (ALT), uric acid, creatine kinase (CK), lactate dehydrogenase (LDH), and thiobarbituric acid reactive substances (TBARS).

**Results:**

Compared to the DC group, the TD group showed body weight gain (↑7.99%, *p* = 0.0153) and attenuated glycemia (↓23.14%, *p* = 0.0008) and total cholesterol (↓31.72%, *p* ≤ 0.0041) associated with diminished reactive species markers in pancreatic (↓45.53%, *p* < 0.0001) and cardiac tissues (↓51.85%, *p* < 0.0001). In addition, compared to DC, TSD promoted body weight recovery (↑15.44%, *p* ≤ 0.0001); attenuated glycemia (↓42.40%, *p ≤* 0.0001), triglycerides (↓39.96%, *p* ≤ 0.001), and total cholesterol (↓28.61%, *p* ≤ 0.05); and attenuated the reactive species markers in the serum (↓26.92%, *p* ≤ 0.01), pancreas (↓46.22%, *p* ≤ 0.0001), cardiac (↓55.33%, *p* ≤ 0.001), and skeletal muscle (↓42.27%, *p* ≤ 0.001) tissues caused by T1DM.

**Conclusion:**

Resistance training associated (and/or not) with the use of *Curcuma longa* attenuated weight loss, the hypoglycemic and hypolipidemic effects, reactive species markers, and T1DM-induced tissue injury.

## Background

Diabetes mellitus (DM) is one of the most serious diseases affecting the population worldwide, and it is estimated that the world’s diabetic population will be 578 million in 2030 and 700 million in 2045 [1]. This epidemic has a higher rate of incidence in both developed and developing countries [2–4]. International guidelines such as the American Diabetes Association and the World Health Organization classify DM into four categories: Type 1 DM (T1DM), Type 2 DM (T2DM), Other Types, and Gestational Diabetes. Type 2 DM is the most prevalent form of the disease, accounting for approximately 90% of the cases in the world’s population [5–7].

Patients with T1DM typically have residual or no insulin production due to loss of functioning of pancreatic beta cells [2, 8, 9]. When not promptly treated with insulin, these patients develop marked hyperglycemia and ketosis with consequent hyperketonemia, proteolysis and lipolysis. Conversely, T2DM is intimately correlated with long-term insulin resistance and compensated hyperinsulinemia, which progresses to T2DM when the insulin response to glucose demands becomes insufficient, leading to insulinopenia with consequent hyperglycemia. At the diagnosis of T2DM, approximately 50% of beta cells are not functioning, in a state of mix of impairments between insulin production and action. In contrast to T1DM, patients affected by T2DM are typically asymptomatic and only develop manifestations either when insulin production becomes vestigial and a ketogenic state is instated or by one of the multiple diabetic complications [2, 8, 9].

Despite the different types of diabetes, overall and individual medical costs and loss of income have been reported to be higher in patients with T1DM compared to T2DM [10, 11]. T1DM is considered to be an inflammatory and autoimmune disorder that results from the infiltration of autoreactive T lymphocytes and the consequent generation of proinflammatory cytokines and the appearance of excessive reactive oxygen species (ROS). As a consequence, increased pancreatic β cell death results in hyperglycemia, which is dependent on exogenous insulin administration, concomitantly with evident glucagon secretion imbalance [12–14].

Pharmacological treatment of diabetes includes insulin and hypoglycemic and anti-hyperglycemic drugs, which will depend on the stage and type of DM. Lifestyle changes are also recommended as nonpharmacological approaches/modalities, among which the most widespread and proven ones are regular exercise and healthy eating [2, 3, 15–17]. In particular, exercise is able to promote beneficial adjustments in aerobic capacity and lipid and glycemic control, as it controls insulin and glucose homeostasis, promotes increased fatty acid oxidation in the muscles, reduces blood glucose concentration, attenuates systemic inflammation and improves immune cell functions [18, 19].

Some authors suggest that resistance training may be recommended as an important therapy in diabetes because it increases skeletal muscle glucose uptake. This is partly due to the action of exercise that promotes increased glucose transporter 4 (GLUT-4) translocation and improved insulin-independent glucose uptake pathway functionality [20]. In addition to this evidence, some authors suggest that physical exercise also improves antioxidant defenses and mitochondrial activity due to the reduction of reactive species [19, 21–23].

On the other hand, the use of herbal medicines to promote glycemic and metabolic homeostasis in pathological conditions such as DM is becoming increasingly popular in the world’s population [24]. In this context, supplementation with turmeric (*Curcuma longa* L.), in different ways, appears to act beneficially on glycemic control; these effects are partly due to an attenuation of insulin resistance and frequently encountered comorbidities in patients with diabetes [25, 26]. Some authors suggest that these effects of turmeric are partly due to the high concentrations of curcumin that have antioxidant action [27–29]. Studies have shown that curcumin also has protective effects, such as increased antioxidant activity of enzymes and mitigation of mitochondrial dysfunction and liver damage [30, 31].

Despite this evidence, some authors showed that the use of high doses of natural supplements at supraphysiological concentrations may result in possible overall risks to health and/or no effects on the whole body, since the safety profile has not been established for the above-recommended dosages [32]. For this reason, it is imperative that personalized antioxidant supplementation may improve performance exercise. This is due, at least in part, to a fine synchronic adjustment of the redox system, as well as other molecular mechanisms that are recruited during exercise adaptation [33].

The present study aimed to evaluate the effects of medium- to high-intensity resistance training associated (or not) with the supplementation of *Curcuma longa* on body weight recovery, blood glucose, lipid profile, reactive species, and muscle damage in Wistar T1DM rats.

## Methods

### Animals

Forty male 3-month-old Wistar rats weighing approximately 250–300 g from the Sector Vivarium of the Intracellular Signaling Research Center of the Federal University of Sergipe were used in this study. They were randomly housed in appropriate conditions – 22 ± 3 °C, 12-h light/dark cycle (300 lx of light), and free access to rodent-specific feed (Labina®) and water ad libitum. The methodology used in the present study were approved by the Ethics Committee on Animal Research of the Federal University of Sergipe (CEPA Protocol 72/18).

### Induction of diabetes mellitus

Experimental DM was induced as described by Santos et al. [34], briefly an solution of 2% aqueous alloxan solution (single dose of 150 mg/kg) (alloxan monohydrate A7413 – Sigma, St. Louis, USA) was injected intraperitoneally into 40 animals. One week after the administration the animals underwent a 24-h fast to enhance the drug’s sensitivity and diabetogenic action with water supply ad libitum. The alloxan administration was conducted and, 30 min after, feed was offered to all groups to prevent hypoglycemia. Blood was collected by caudal puncture for the blood glucose by means of an Accu-Chek Go glucometer (Roche Diagnostics GmbH, D-68298, Mannheim, Germany) test 72 h after of induction. Only animals with fasting blood glucose of 200 mg/dL or higher were included in the study, starting in the treatment and resistance training protocol (RTP) protocol.

### Resistance training protocol

RTP was performed by means of a flexion-extension (which involves the soleus, extensor digitorum longus, and gastrocnemius muscular groups) using a squat machine. The animals wore a jacket that connected them to a articulated 35-cm-long wooden bar where the loads were allocated. During the routine, the rats were sited on their back legs, according to the method by Tamaki et al. [35] and adapted by Santos et al. [34].

All animals used the equipment for one week in order to get used to it and also received electrostimulation. Afterwards, the DT and TSD animals underwent the training protocol of 3 × 10 repetitions, with intervals of 60s between the sets, at an intensity of 70% of the load that was established by the one-repetition maximum (1RM) test [35]. The RT was performed three times a week for four weeks every other day [34, 35]. The load used in the training routine was adjusted every two weeks following a new 1RM test. The DC and SD animals underwent the same methodology but with no load and 0% intensity (Table 1). The electrical stimulation (20 V/0.3 s in duration, 3-s interval) was applied to using electrodes (ValuTrode, Model CF3200, Axelgaard, Fallbrook, CA, USA) fixed to their tail by an electrostimulator (BIOSET, Physiotonus Four, Model 3050, Rio Claro, SP, Brazil). The load used was low and did not induce changes in the stress predictors [36].

**Table 1:** Resistance training protocol

Week	Intensity (%)	Days in the week ^a^	Number of series	Repetitions (n)	Interval (seconds)
1st	70	3	3	10	60
2nd	70	3	3	10	60
3rd	70	3	3	10	60
4th	70	3	3	10	60

Model proposed by Tamaki et al. [35] and ^a^ adapted by Santos et al. [34]

### Experimental groups

The body weight measurement and blood glucose were checked once each week. *Curcuma longa* supplementation and the RTP protocol were performed three times per week. The animals were divided into four groups (*n* = 7–10 for each group): 1) diabetic control group (DC): diabetic and sedentary animals treated with vehicle (distilled water, oral) + electrostimulation with no load on the apparatus; 2) trained diabetics (TD): diabetic animals treated with vehicle and submitted to RTP; 3) supplemented diabetics (SD): diabetic animals treated with *Curcuma longa* L. extract (200 mg/kg, orally) + electrostimulation with no load on the apparatus; 4) trained supplemented diabetics (TSD): diabetic animals treated with *Curcuma longa* L. extract (200 mg/kg orally) and submitted to RTP.

### Supplementation

Supplementation consisted of administering *Curcuma longa* extract from the manufacturer Florien, São Paulo, Brazil, with Internal Lot #: 18A20-B018–028830 and Manufacturer Lot #: CJH-A-706694. The extract was administered at a dose of 200 mg/kg three times per week, always at the same time after the RTP protocol by oral gavage using a rodent-specific stainless-steel cannula with a rounded end not to cause any injury.

### Sample collection

All the animals were euthanized at the end of the study (30 days) with a solution of ketamine (100 mg/kg) and xylazine (10 mg/kg) administrated intraperitoneally, and then, their blood and organs (pancreas, heart, liver, and gastrocnemius muscle) were collected, weighed, and stored for later analysis.

### Determination of serum biochemical markers

Blood was centrifuged at 800 x *g* for 15 min at 4 °C, and the serum was stored at − 80 °C. The serum was used to measure the concentrations of triglycerides, total cholesterol and HDL cholesterol (HDL), alanine aminotransferase (ALT), aspartate aminotransferase (AST), creatine kinase (CK), lactate dehydrogenase (LDH), and uric acid according to the manufacturer’s procedures (Labtest®, Lagoa Santa, Minas Gerais, Brazil). The reactive species markers were also evaluated as follows: lipoperoxidation was evaluated by thiobarbituric acid reactive substances (TBARS) following the method proposed by Lapenna et al. [37].

### Determination of tissue reactive species markers (TBARS)

The organs were washed three times in a potassium chloride solution (1.15% KCl) and homogenized (1:5 p/v) with KCl solution, phenylmethylsulfonyl fluoride (PMSF 100 m.mol-1), and Triton solution (10%). Immediately thereafter, the homogenates were centrifuged at 3000 x *g* for 10 min at 4 °C, and the supernatant was stored at − 80 °C for the analysis of reactive species markers (TBARS). The results were expressed per gram of tissue.

### Statistical analysis

All the statistical analysis was performed in the software Graph Pad Prism version 5.0 and the outcome is presented as the mean ± standard deviation (X ± SD) as a result of a triplicate analysis (to TBARS samples). Firstly, the data was evaluated about its normality, using the Shapiro Wilk test, afterwards they were statistically evaluated among groups by one-way analysis of variance (ANOVA one way) and post hoc Bonferroni test. Two of the groups were statistically evaluated by using the *t*-test. The statistically significant difference between the samples adopted was *p* < 0.05.

## Results

The diabetic animals of all groups presented polyuria, polydipsia, and polyphagia (data not shown) in the 72 h after alloxan induction, and the symptoms remained until the last day of the experiment.

**Fig. 1:**
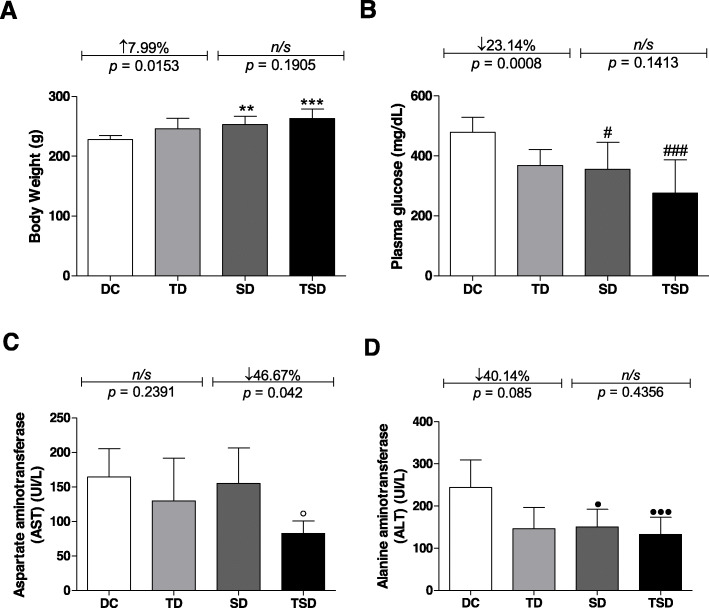
Analysis of body weight, fasting plasma glucose, aspartate aminotransferase, and alanine aminotransferase of the diabetic animals group (DC) (white bar); trained diabetics (TD) group submitted to resistance training for four weeks (light gray bar); supplemented diabetics (SD) group receiving 200 mg/kg body three times per week for four weeks (dark gray bar); and trained and supplemented diabetics (TSD) group submitted to *Curcuma longa* supplementation and the resistance training program simultaneously (black bar). Body weight (**a**), fasting plasma glucose (**b**), aspartate aminotransferase (AST) (**c**) and alanine aminotransferase (ALT) (**d**). Data represent the mean ± standard deviation of the mean. One-way analysis of variance (ANOVA one way) and post hoc de Bonferroni tests were used. Letters on the bars represent the significant difference by one-way ANOVA followed by Bonferroni’s test among the groups as follows: body weight (^**^p ≤ 0.01 for DC vs. SD; ^***^p ≤ 0.001 for DC vs. TSD), glucose (^#^*p* ≤ 0.05 for DC vs. SD; ^###^*p* ≤ 0.001 for DC vs. TSD), AST (*p* ≤ 0.05 for DC vs. TSD), and ALT (^●^*p* ≤ 0.05 for DC vs. SD; ^●●●^*p* ≤ 0.001 for DC vs. TSD). For the DC vs. TD and SD vs. TSD groups, Student’s t-test was used. ns = no significant difference. *n* = 7–8 in all experimental groups

Figure 1a represents the results regarding the body weight of the groups at the end of the experimental period. The animals belonging to the TD, SD, and TSD groups presented a body weight increase of 7.99% (TD = 246.01 ± 17.5 g, *p* = 0.0153), 10.97% (SD = 252.9 ± 14.01 g, *p* ≤ 0.01), and 15.45% (TSD = 263.1 ± 15.73 g, *p* ≤ 0.001), respectively, when compared to the diabetic group without any intervention (DC = 227.9 ± 6.556 g). When comparing the TD, SD, and TSD groups, there were no significant differences among them.

Figure 1b represents the plasma glucose of the different experimental groups. The TD group at the end of the experiment showed a reduction of 23.14% (368.0 ± 53.22 mg/dL, *p* = 0.0008) compared to the DC group (478.8 ± 50.06 mg/dL). This reduction was similar in the SD (355.0 ± 90.93 mg/dL) and TSD (275.8 ± 111.4 mg/dL) groups, which were approximately 25.86% (*p* ≤ 0.05) and 42.40% (*p* ≤ 0.001) lower, respectively, when compared with the DC group. When comparing the TD, SD and TSD groups, there were no significant differences among them.

The concentrations of aspartate aminotransferase (AST) (Fig. 1c) in the DC (164.4 ± 40.85 UI/L), TD (129.7 ± 61.86 UI/L), and SD (155.1 ± 51,35 UI/L) groups were similar. The results show that there was a 49.69% (*p* ≤ 0.05) and 46.67% (*p* = 0.042) reduction in the TSD group (82.71 ± 18.03 UI/L) when compared to the DC and SD groups, respectively. When comparing the TD and TSD groups, there was no significant difference between them.

In Fig. 1d**,** the plasma alanine aminotransferase (ALT) content was reduced by 40.14% (*p* = 0.085) in the TD (146.1 ± 50.46 UI/L), 38.30% (p ≤ 0.05) in the SD (150.6 ± 41.63 UI/L), and 45.60% (*p* ≤ 0.001) in the TSD (132.9 ± 40.54 UI/L) groups compared with the DC group (244.1 ± 65.42 UI/L (*p* < 0.05). When comparing the TD, SD, and TSD groups, there were no significant differences among them.

**Fig. 2:**
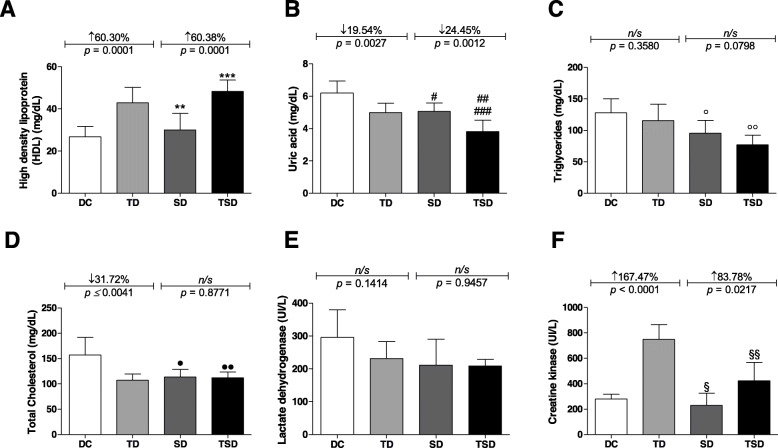
Analysis of lipid profile, lactate dehydrogenase, and creatine kinase of the diabetic animals group (DC) (white bar); trained diabetics (TD) group submitted to resistance training for four weeks (light gray bar); supplemented diabetics (SD) group receiving 200 mg/kg body three times per week for four weeks (dark gray bar); and trained and supplemented diabetics (TSD) group submitted to *Curcuma longa* supplementation and the resistance training program simultaneously (black bar). High-density lipoprotein (HDL) (**a**), uric acid (**b**), triglycerides (**c**), total cholesterol (**d**), lactate dehydrogenase (**e**), and creatine kinase (**f**). Data represent the mean ± standard deviation of the mean. Letters on the bars represent the significant difference by one-way ANOVA followed by Bonferroni’s test among groups as follows: high-density lipoprotein (HDL) (^**^p ≤ 0.01 for SD vs. TD; ^***^p ≤ 0.001 for DC vs. TSD), uric acid (^#^*p* ≤ 0.01 for DC vs. SD; ^##^*p* ≤ 0.001 for DC vs. TSD; ^###^*p* ≤ 0.01 for TD vs. TSD), triglycerides (^°^*p* ≤ 0.01 for DC vs. TSD; ^°°^*p* ≤ 0.05 for TD vs. TSD), and creatine kinase (^§^ ≤ 0.001 for TD vs. SD; ^§§^ ≤ 0.001 for TD vs. TSD). The DC vs. TD and SD vs. TSD groups were analyzed using Student’s t-test. ns = no significant difference. n = 7–8 in all experimental groups

Regarding HDL cholesterol (Fig. 2a), there was a 60.30% (*p* = 0.0001) increase in the TD group (42.88 ± 7.28 mg/dL) and 80.37% (*p* ≤ 0.001) in the TSD group compared to the DC group (26.75 ± 4.89 mg/dL). There was also an increase of 60.38% (*p* ≤ 0.0001) in the TSD group (48.25 ± 5.50 mg/dL) compared to the SD (30.0 ± 7.87 mg/dL) group. When the values of HDL cholesterol in the SD group were compared to the TD group, there was an increase of 30.04% (*p* ≤ 0.001).

For the concentrations of uric acid (Fig. 2b), there was a reduction of 19.54% (*p* = 0.0027) in the TD group (4.99 ± 0.584 mg/dL), 18.34% (*p* ≤ 0.01) in the SD group (5.063 ± 0.5263 mg/dL), and 38.31% (*p* ≤ 0.001) in the TSD group (3.825 ± 0.6902 mg/dL) compared to the DC (6.20 ± 0.740 mg/dL) group. The results show that there was a 24.45% (*p* = 0.0012) reduction in the TSD group compared to the SD group. When the uric acid levels of the TSD group were compared to those of the TD group, there was a reduction of 23.34% (*p* ≤ 0.01).

Figure 2c shows the results for the triglyceride concentration where no significant difference occurred between the DC (127.9 ± 22.30 mg/dL) and TD (115.4 ± 26.45 mg/dL) groups, nor in the SD (95.43 ± 20.33 mg/dL) group. However, the results show that there was a reduction of 39.96% (*p* ≤ 0.01) and 23.14% (*p* ≤ 0.05) in the TSD (77.14 ± 15.03 mg/dL) group when compared to the DC and TD groups, respectively.

Total cholesterol fractions (Fig. 2d) were reduced by 31.72% (*p* = 0.0041), 27.91% (*p* ≤ 0.05), and 28.61% (*p* ≤ 0.05) in the TD (107.4 ± 12.09 mg/dL), SD (113.4 ± 15, 46 mg/dL), and TSD (112.3 ± 11.28 mg/dL) groups, respectively, compared to the DC group (157.3 ± 34.47 mg/dL). However, no significant differences occurred among the TD, SD, and TSD groups.

The lactate dehydrogenase concentration was evaluated (Fig. 2e), and there were no significant differences displayed among the DC (295.8 ± 84.18 UI/L), TD (231.7 ± 51.05 UI/L), SD (211.2 ± 79.32 UI/L), and TSD (208.8 ± 20.36 UI/L) groups.

Figure 2f illustrates the plasma creatine kinase concentration. A decrease of 43.58% (*p* = 0.0016) was detected in the TSD group (422,5 ± 145.1 UI/L) compared to the TD group (748.9 ± 116 UI/L). However, no significant differences occurred between the DC group (280.0 ± 38.24 UI/L, *p* = 0.2598) and the SD group (229.9 ± 95.25 UI/L).

**Fig. 3:**
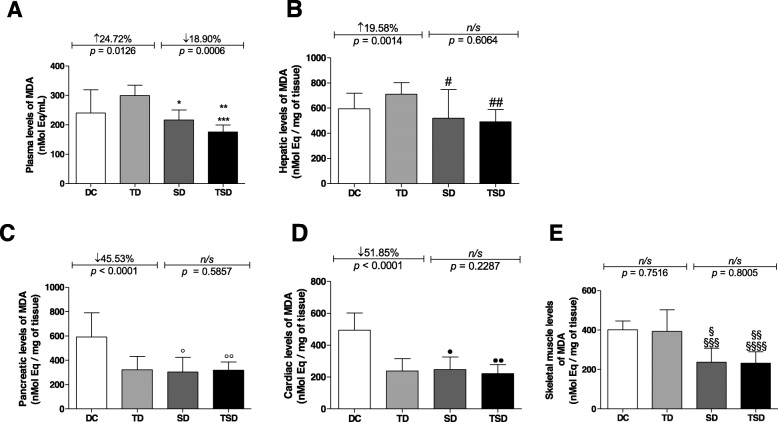
Analysis of tissue damage markers of the diabetic animals group (DC) (white bar); trained diabetics (TD) group submitted to resistance training for four weeks (light gray bar); supplemented diabetics (SD) that received 200 mg/kg body three times per week for four weeks (dark gray bar); and trained and supplemented diabetics (TSD) group submitted to *Curcuma longa* supplementation and the resistance training program simultaneously (black bar). Plasma TBARS (**a**), liver tissue TBARS (**b**), pancreatic tissue TBARS (**c**), heart tissue TBARS (**d**), and muscle tissue TBARS (**e**). Data represent the mean ± standard deviation of the mean. Letters on the bars represent the significant difference by one-way ANOVA followed by Bonferroni’s test among groups as follows: plasma TBARS (^*^p ≤ 0.01 for SD vs. TSD; ^**^p ≤ 0.01 for TD vs. SD; ^***^p ≤ 0.01 for TD vs. TSD), liver tissue TBARS (^#^*p* ≤ 0.001 for TD vs. SD; ^##^*p* ≤ 0.01 for TD vs. TSD), pancreatic tissue TBARS (^°^*p* ≤ 0.001 for DC vs. SD; ^°°^*p* ≤ 0.001 for DC vs. TSD), heart tissue TBARS (^●^*p* ≤ 0.001 for DC vs. SD; ^●●^*p* ≤ 0.001 for DC vs. TSD), and muscle tissue TBARS (^§^ ≤ 0.001 for DC vs. SD; ^§§^ ≤ 0.001 for DC vs. TSD; ^§§§^ ≤ 0.001 for TD vs. SD; ^§§§§^ ≤ 0.0010 for TD vs. TSD). The DC vs. TD and SD vs. TSD groups were analyzed using Student’s t-test. ns = no significant difference. n = 7–8 (in triplicate) in all experimental groups

Regarding plasma reactive species markers (Fig. 3a), the TD group (299.7 ± 35.12 nMolEq/mL, *p* = 0.0126) showed a 24.72% increase in the concentration of thiobarbituric acid reactive substances (TBARS) compared to the DC group (240.3 ± 78.85 nMolEq/mL). There was no significant difference between the DC and SD groups (216.5 ± 33.55 nMolEq/mL); however, the SD and TSD (175.6 ± 23.40 nMolEq/mL) groups showed a decrease of 27.76% (*p* ≤ 0.01) and 41.41% (*p* ≤ 0.001) in the TBARS concentration, respectively, when compared to the TD group. When the value of the concentration of thiobarbituric acid reactive substances (TBARS) of the TSD group was compared with that of the DC and SD groups, there was a reduction of 26.92% (*p* ≤ 0.01) and 18.90% (*p* = 0.0006), respectively.

Figure 3b illustrates the TBARS marker in the liver tissue; there was a 19.58% (*p* = 0.0014) increase in the TD group (710.8 ± 93.28 nMolEq/gram tissue) compared to the DC group (594.4 ± 123.9 nMolEq/gram tissue). However, no significant differences were found between the DC, SD (520.0 ± 227.3 nMolEq/gram tissue), and TSD (492.0 ± nMolEq/gram tissue) groups. There was a similar effect between the SD and TSD (*p* = 0.06064) groups, but the SD and TSD groups showed a reduction of 26.84% (*p* ≤ 0.001) and 30.78% (p ≤ 0.01), respectively, in the TBARS concentrations when compared with the TD group. The effect in the TSD group, however, was similar to that of the DC group.

Figure 3c shows the concentration of TBARS in the pancreas, where a 45.53% (*p* < 0.0001) reduction in TBARS was observed in the TD group (322.3 ± 110.6 nMolEq/gram tissue) compared to the DC group (591.7 ± 198.5 nMolEq/gram tissue). The comparison between the SD (319.8 ± 145.5 nMolEq/gram tissue) and TSD (324.0 ± 75.24 nMolEq/g tissue) groups did not differ (*p* = 0.5857). However, the SD and TSD groups showed a reduction of 48.84% (*p* ≤ 0.001) and 46.22% (p ≤ 0.001), respectively, in the TBARS concentrations when compared to the DC group. The effects in the TD, SD, and TSD groups were similar to each other.

The results of TBARS in cardiac tissue are depicted in Fig. 3d. In the TD (237.9 ± 78.45 nMolEq/gram tissue), SD (246.9 ± 79.63 nMolEq/gram tissue), and TSD (220.7 ± 57.23 nMolEq/gram tissue) groups, reductions of 51.85% (*p* ≤ 0.001), 50.03% (*p* ≤ 0.001), and 55.33% (*p* ≤ 0.001), respectively, were observed when compared with the DC group (494.1 ± 108.2 nMolEq/gram tissue). There were no significant differences when comparing the TD, SD, and TSD groups with each other.

The TBARS concentrations in skeletal muscle tissue (Fig. 3e) were similar in the DC (401.2 ± 44.57 nMolEq/gram tissue) and TD (392.9 ± 110.5 nMolEq/g tissue) groups (*p* = 0.7516); the SD (236.7 ± 70.84 nMolEq/gram tissue) and TSD (231.6 ± 57.62 nMolEq/gram tissue) groups also showed no significant difference between them (*p* = 0.8005). However, the SD group showed a reduction of 39.76% (p ≤ 0.001) compared to the TD group (Fig. 3f). The results show that there was a reduction of 41.05% (p ≤ 0.001) and 42.27% (p ≤ 0.001) in the TSD group when compared to the TD and DC groups, respectively. There was also a reduction of 41.00% in the SD group compared to the DC (*p* < 0.001) group.

## Discussion

The present study aimed to evaluate moderate- to high-intensity RTP-associated *Curcuma longa* supplementation and its effects on weight gain, recovery, glycemic control, muscle damage, and reactive species markers in alloxan-induced type-1 diabetic rats. The use of alloxan in an experimental model causes a similar picture as in some humans with type 1 diabetes without blood glucose control, involving symptoms such as polydipsia and polyphagia and a marked reduction in body weight [34, 38, 39].

The animals from the DC group showed a significant reduction in body weight throughout the experiment. This symptom is due to the effects of untreated T1DM in chronic conditions (without insulin therapy), which among its effects is evidence of exacerbated protein and lipid catabolism associated with glycosuria and polyuria [40, 41]. However, the TD, SD, and TSD groups presented attenuation of body weight reduction. The beneficial effect of *Curcuma longa* supplementation (SD groups) on body weight was similar to the results from other authors [42].

According to the American Diabetes Association [43], glycemic alterations over a long period of time cause numerous metabolic dysfunctions, among which the most common are autonomic peripheral neuropathies, retinopathy, ketoacidosis, and nonketotic hyperosmolar syndrome.

In patients with T1DM, the plasma glucose concentration must be maintained under conditions close to the ideal values (when the baseline result is equal to or above 126 mg/dL and the oral glucose tolerance test is at or above 200 mg/dL, the disease is proven) to attenuate the development of metabolic diseases such as retinopathy, limb amputation, and dyslipidemia [2, 43]. Supplementation with *Curcuma longa* for 21 days in supplemented type 1 diabetic rats was able to promote a marked reduction in the blood glucose concentration [44]. This effect was similar to our study, which showed that supplementation of turmeric at a dosage of 200 mg/kg body weight can attenuate plasma glucose.

Both resistance training and *Curcuma longa* supplementation showed a hypoglycemic effect throughout the treatment, as there was a reduction in the blood glucose in the experimental groups TD, SD, and TSD. Although the RTP protocol alone promoted a reduction in blood glucose, it was even greater with the combination of RTP associated with *Curcuma longa* supplementation. However, no differences were observed when comparing the TD, SD, and TSD groups.

The glucose-lowering effects of CL supplementation and exercise in the complete absence of any residual insulin observed in the present study may elicit novel insulin-independent glucose-lowering pathways in T1DM and should be a matter of further investigation.

RTP is able to provide beneficial effects on patients with diabetes, as it can promote increased glucose uptake in skeletal muscles. This effect is partly due to the possibly increased GLUT-4 translocation and the beneficial adjustment of the insulin-independent glucose uptake pathway [16–19, 45]. Curcumin is effective in the prevention and control of diabetes since it helps to reduce the concentration of glycated hemoglobin and, consequently, controls plasma glucose by mechanisms that are not yet fully understood [46–48].

The severe hyperglycemia in uncontrolled T1DM results in a low input of tissue and cellular energy substrate. As a consequence, it causes suppression of ATP genesis and the activation of pathways involved in the formation of ROS [49, 50].

Some authors suggested that when glucose and free fatty acids are increased in the blood, they may activate molecular mechanisms in different cellular types. This scenario involves electron transport overload, increased formation of metabolic byproducts, electron leak, ROS generation, and upregulation of inflammatory signaling [9]. We hypothesize that supplementation with *Curcuma longa* may play an important role in ROS production by mechanisms that are not yet known.

Experimental diabetes also shows hypertriglyceridemia in animals treated with alloxan [51]; during T1DM, untreated animals showed enhanced fatty acid release by adipose tissues during ketosis. In our studies, the results were similar; sedentary diabetic animals (DC) showed increased concentrations of triglycerides and total cholesterol compared to SD and TSD, respectively. These effects are partly due to disease progression caused by the imbalance of some of the macrovascular and microvascular risk factors [43].

Thus, supplementation with RTP-associated *Curcuma longa* for four weeks has been shown to effectively improve the lipid profile of diabetic animals, resulting in reductions in the total cholesterol and triglyceride concentrations and an increase in HDL. Our results corroborate the results of Su et al. [52], who used curcumin supplementation for eight weeks and found a reduction in the concentration of high-intensity lipoprotein cholesterol (LDL-C) and triglycerides and an increase in the concentration of high-intensity lipoprotein (HDL-C). In addition, this effect of *Curcuma longa* (CL) supplementation on the body is maintained over time. According to another study, supplementation with 1000 mg/day of *Curcuma longa* for 12 weeks is also able to promote a reduction in total serum cholesterol (TC), LDL-C, and triglycerides and cause an increase in HDL-C [46]. Despite this evidence, more studies are necessary to clarify this effect of CL supplementation on the lipid profile.

In healthy organisms, more specifically in their cells, the high concentration of free radicals is temporary because the body is able to activate the antioxidant defense system. However, a constant metabolic imbalance between the increased concentration of reactive species and the decreased concentration and/or activity of antioxidant molecules characterizes an organic and metabolic condition called oxidative stress, which is associated with numerous pathologies, including diabetes mellitus [53].

Studies have shown a significant increase in reactive species associated with physical exercise with maximal and supramaximal intensities [54–57]. Exercise-induced tissue stress causes the recruitment and migration of leukocytes and cells from the immune system to the damaged area, which is proportional to the exercise intensity [58, 59].

Some authors suggest that physical exercise is capable of inducing tissue damage; these effects are partly due to reactive species markers caused by the increased concentration of CK, LDH, and malonaldehyde markers [34, 60]. However, it is noteworthy that this increase will depend on the mode, intensity, and duration of the physical training. In addition, the oscillation of these parameters is an adaptive response of the body to moderate exercise and is beneficial to health [61].

Reactive species can be triggered by a number of factors, including increased metabolism of prostanoids, xanthine oxidase, and NADPH oxidase enzymes, oxidation of purine bases and iron-containing proteins, and disruption of Ca^2+^ homeostasis [62, 63].

The present research showed that moderate- to high-intensity resistance training induced tissue injury when there was a significant increase in CK and plasma LDH concentrations in the group of trained diabetic animals. However, we were not able to determine whether these changes remained after the training had ended. In the present research, the animals from the SD and TSD groups that were treated with *Curcuma longa* supplementation and underwent resistance physical training presented a significant reduction in the markers of CK, ALT, and uric acid; however, we did not observe reductions in the AST and LDH concentrations.

Corroborating these results with an experimental model, in the study by Tanabe et al. [64], where oral supplementation of Curcuma 180 mg was used in humans for seven days before and after isokinetic eccentric exercise, positive effects were noted on the inflammatory and muscle damage markers, presenting a reduction in CK activity with supplementation between 3 and 6 days before exercise and 5 to 7 days after exercise. Therefore, these results demonstrate that *Curcuma longa* supplementation associated with physical exercise can be effective in mitigating reactive species production.

In addition, tissue damage caused by high-intensity RTP can be reduced with the use of medicinal plants containing polyphenols and antioxidants such as vitamins A, E, and C [65–69].

Supplementation with antioxidants is capable of modulating the redox state of cells and counteracting the deleterious effects caused by ROS, with the possibility of reversing and/or attenuating lipid peroxidation caused by reactive species [70].

DM is a condition associated with increased free radicals due to the increased production of the TBARS marker in various tissues [71, 72]. Thus, this research aimed to elucidate the effects of *Curcuma longa* supplementation and its possible protective effect on reactive species markers in organs such as the pancreas, liver, heart, and skeletal muscle of diabetic animals.

In the liver tissue, *Curcuma longa* supplementation reduced the TBARS concentrations, showing positive effects on lipid oxidation when compared to the group of diabetic animals without any treatment. Reductions in the TBARS concentrations in the SD and TSD groups confirmed that RTP and the supplementation results in the control of reactive species markers, which may explain the decrease in the plasma glucose concentration in these experimental groups.

The pancreas is an organ susceptible to oxidative stress and damage caused by the increased concentration of reactive species under altered metabolic conditions [73, 74]. In general, type 1 diabetes (T1DM) is characterized by inflammation of the pancreatic islets, associated with increased free radical species, proinflammatory cytokines, and immune cell migration with specific β-cell antibodies, leading to β-cell dysfunction and cellular death [8, 9]. Consequently, enhanced islet α-cell activity leads to a hyperglucagonemia state that enhances hepatic glucose production by the action of this hormone [2, 9, 75]. In our study, we found a decrease in the concentration of TBARS in the pancreas in the TD, SD, and TSD groups. Thus, both physical training and supplementation were able to attenuate the deleterious effects of diabetes on this organ.

Both T1DM and T2DM present as a highly inflammatory and oxidative pathology, presenting a direct relation to cardiovascular events. In nondrug therapy, the use of antioxidants and phenolic compounds shows positive effects on redox action and has a protective effect on the heart tissue and prevents damage to it [76–78]. In the present study, both resistance training and supplementation and their combination demonstrated protective effects against the damage caused by reactive species to the cardiac tissue in T1DM.

Thus, supplementation with *Curcuma longa* associated with RTP was able to provide body weight recovery, reduce glycemic rates, and attenuate reactive species production and tissue damage caused by hyperglycemia when compared to controls or physical activity alone.

## Conclusion

In summary, the use of *Curcuma longa* associated with the RTP protocol is able to attenuate weight loss in chronic metabolic conditions caused by T1DM, which is associated with reduced blood glucose and lipid parameters. These effects are partly due to the reduced activity of some reactive species markers associated with moderate- to high-intensity resistance training, and the content of some T1DM-induced tissue injury marker enzymes were significantly reduced. Therefore, physical training associated with *Curcuma longa* represents a potential nonpharmacological therapeutic alternative in the treatment of T1DM.

Despite these results, it is certainly not sufficient. Lipid peroxidation is a complex process, as mentioned even by other authors [78, 79], and we suggest that further investigations should be made to detect what molecule(s) is involved in lipid peroxidation.

We hypothesize that even without insulin use for T1DM, exercise alone or in association with CL was able to promote adjustments that resulted in a reduction in blood glucose levels, attenuation of the activation of systemic inflammation and attenuation of the recruitment of immune cells, typically caused by uncontrolled T1DM. Glucose improvement irrespective of insulin and under undetectable insulin levels is a novel mechanism that is possibly mediated by reactive oxygen species-dependent pathways [8, 9]. However, these findings as well as the underlying mechanisms should be investigated.

In addition, we advocate that the influence of diet on physical activity parameters is not discarded, as is well documented by other authors [80, 81], and it is necessary in future studies to analyze the interaction of diet on physical activity parameters considering individual characteristics to guarantee the correct ingestion of antioxidant supplementation and their beneficial effects on the whole body [33].

## Data Availability

The datasets used and/or analysed during the current study available from the corresponding author on reasonable request.

## Abbreviations

DM: Diabetes mellitus (DM)
CL: *Curcuma longa*;
T1DM: Type 1 diabetes mellitus
DC: Sedentary diabetic rats
TD: Diabetic rats that underwent a 4-week resistance training protocol
SD: CL-supplemented diabetic rats
TSD: Supplemented diabetic rats that underwent the same conditions as above
HDL: High-density lipoprotein
ALT: Alanine aminotransferase
AST: Aspartate aminotransferase
CK: Creatine kinase
LDH: Lactate dehydrogenase
TBARS: Thiobarbituric acid reactive substances
ROS: Reactive oxygen species
GLUT-4: Glucose transporter 4.
CEPA: Ethics Committee on Animals
RTP: Resistance training protocol
KCL: Potassium chloride solution
ATP: Adenosine triphosphate
NADPH: Chemically reduced form of NADP
